# Expansion of the Milan criteria without any sacrifice: combination of the Hangzhou criteria with the pre-transplant platelet-to-lymphocyte ratio

**DOI:** 10.1186/s12885-016-3028-0

**Published:** 2017-01-05

**Authors:** Weiliang Xia, Qinghong Ke, Hua Guo, Weilin Wang, Min Zhang, Yan Shen, Jian Wu, Xiao Xu, Sheng Yan, Jun Yu, Mangli Zhang, Shusen Zheng

**Affiliations:** 1 Division of Hepatobiliary and Pancreatic Surgery, Department of Surgery, First Affiliated Hospital, School of Medicine, Zhejiang University, Qingchun Road 79, Hangzhou, 310003 China; 2 Key Laboratory of Combined Multi-organ Transplantation, Ministry of Public Health, First Affiliated Hospital, School of Medicine, Zhejiang University, Qingchun Road 79, Hangzhou, 310003 China

**Keywords:** Hangzhou criteria, Liver transplantation, Hepatocellular carcinoma, Platelet-to-lymphocyte ratio

## Abstract

**Background:**

The Hangzhou criteria expand the Milan criteria safely and effectively in selecting hepatocellular carcinoma (HCC) candidates for liver transplantation (LT), but some patients exceeding the Milan but fulfilling the Hangzhou criteria still show poor outcomes due to early tumor recurrence. In this study, the platelet-to-lymphocyte ratio (PLR) was employed to differentiate high-risk tumor recurrence recipients, and a new method combining PLR and the Hangzhou criteria was established.

**Methods:**

The clinical data of 343 LT for HCC were retrospectively analyzed. Receiver operating characteristic (ROC) analysis was used to determine the PLR cut-off value to stratify patients exceeding the Milan but fulfilling the Hangzhou criteria. The recurrence-free survival (RFS) of recipients was compared after stratification. The Hangzhou criteria & PLR method was proposed and its feasibility was validated by ROC analysis.

**Results:**

PLR 120 was the most significant cut-off value when comparing RFS of patients exceeding the Milan but fulfilling the Hangzhou criteria. After stratification, the 1-, 3-, and 5-year RFS of patients exceeding the Milan but fulfilling the Hangzhou criteria with PLR < 120 were 84.2%, 73.3%, and 73.3%, respectively, comparable with 85.7%, 73.9%, and 72.8%, respectively, in patients fulfilling the Milan criteria (*P* = 0.885). Patients exceeding the Milan but fulfilling the Hangzhou criteria with PLR ≥ 120 showed poor outcomes, which were similar in patients exceeding the Hangzhou criteria; 1-, 3-, and 5-year RFS were only 37.5%, 12.5%, and 12.5% vs. 32.3%, 17.6%, and 15.1%, respectively (*P* = 0.887). ROC analysis demonstrated that the ROC area of the Hangzhou criteria & PLR method was 0.768 for RFS. Multivariate analysis confirmed that PLR ≥ 120 was independently associated with RFS of patients exceeding the Milan but fulfilling the Hangzhou criteria.

**Conclusions:**

The Hangzhou criteria combined with the pre-transplant PLR can accurately exclude high-risk tumor recurrence recipients; this approach expands the Milan criteria effectively without any sacrifice.

**Electronic supplementary material:**

The online version of this article (doi:10.1186/s12885-016-3028-0) contains supplementary material, which is available to authorized users.

## Background

Hepatocellular carcinoma (HCC) ranks fifth in morbidity, and third in mortality of cancer in the world [1]. In 2012, the estimated number of new liver malignancy and deaths were 782,500 and 745,500, respectively. Unfortunately, about half of the new cases and deaths of liver cancer happened in China [2]. Liver transplantation (LT) provides the best prognosis for well-selected HCC patients by removing both the tumor and the underlying carcinogenic liver. In the early period, the outcome of LT for HCC was disappointing because of the high tumor recurrence rate of about 30–40% [3, 4]. This situation has changed dramatically since the introduction of the Milan criteria (a solitary HCC nodule 5.0 cm or less in diameter or no more than 3 tumor nodules and each 3.0 cm or less in diameter, without tumor invasion of blood vessels or lymph nodes) [5]. In subsequent studies, it was shown that the selection criteria should not be as restrictive as the Milan criteria, and a number of extended criteria have been proposed, such as the University of California San Francisco (UCSF) criteria by Yao et al. in 2001 [6], the Hangzhou criteria (total tumor diameter ≤ 8 cm; or total tumor diameter > 8 cm, with histopathologic grade I or II and pre-operative AFP level ≤ 400 ng/mL, simultaneously) by Zheng et al. in 2008 [7], and the up-to-seven criteria by Mazzaferro et al. in 2009 [8], respectively. After expansion, increased numbers of eligible HCC patients were provided the chance to be transplanted, with an increase of 16.3% by the UCSF criteria and up to 51.5% by the Hangzhou criteria in the Chinese population [9]. Although there is no statistically significant difference in post-transplant survival between the expanded criteria and the Milan criteria, a mathematic decrease in survival rates was shown in patients exceeding the Milan but fulfilling the expanded criteria compared with those fulfilling the Milan criteria [6, 7, 9]. This situation indicates that there are always exceptional patients exceeding the Milan but fulfilling the expanded criteria who will not benefit from LT, so it is important to differentiate these high-risk tumor recurrence patients from the waiting list.

Recently, systemic inflammation has been shown to be related to a poor prognosis and increased tumor progression. As a marker of the systemic inflammatory response, the platelet-to-lymphocyte ratio (PLR) has been shown to be a prognostic factor for various tumors [10, 11]. Our previous study also identified that an elevated PLR predicted increased HCC recurrence rates after LT [12]. In this study, we aimed to investigate the value of PLR in differentiating those high-risk tumor recurrence candidates who exceed the Milan but fulfill the Hangzhou criteria and further establish a new method combining the Hangzhou criteria and pre-transplant PLR to precisely select HCC patients for LT.

## Methods

### Patients

A total of 343 patients who received LT for HCC were enrolled in this retrospective study, and all the HCC developed on the background of liver cirrhosis, which was confirmed by pathology of the explanted liver. The exclusion criteria were (1) recipient age less than 18 years, (2) patients who died during the first month after LT, (3) recipients without clinical data and follow-up data, and (4) patients with pre-transplant sepsis, hypersplenism, or massive gastrointestinal tract bleeding. All the LT were performed at the first affiliated hospital, School of Medicine, Zhejiang University, between January 2003 and December 2013.

Ethical approval was obtained from the Committee of Ethics in Biomedical Research of Zhejiang University and conformed to the ethical guidelines of the Declaration of Helsinki. Written informed consent was obtained from all participants.

### Study design and data collection

The complete blood count was performed every week or as necessary before LT; the PLR was calculated as the ratio of the platelet count to the lymphocyte count according to the complete blood count performed within one month before LT; if more than one set of measurements were available for a given patient, only the lowest PLR value was used.

The diagnosis of HCC and tumor-related characteristics including tumor number, tumor size, macrovascular invasion, microvascular invasion, and tumor cell differentiation grading were evaluated based on the pathological findings. The judgment regarding fulfillment of the Milan or the Hangzhou criteria was based on a pathological examination of explanted livers.

The recipients’ clinical data including age, gender, model of end-stage liver disease (MELD) score, hepatitis B virus (HBV) infection status, and transplantation type (living donor liver transplantation [LDLT] or deceased donor liver transplantation [DDLT]) were collected. Pre-transplant treatment for HCC was also recorded, i.e. surgical resection and interventional therapies including transarterial chemoembolization, thermal ablation, and percutaneous ethanol injection.

### Follow-up

All transplanted recipients were followed up. Screening for tumor recurrence was performed by α-fetoprotein (AFP) measurement and ultrasonography every month during the first six months and performed every two months during the second six months. In subsequent years, the patients received examinations every three to six months or when necessary. Thoracoabdominal computed tomography was performed every six months or when necessary. Bone scan or positron emission tomography was carried out in cases of suspected HCC recurrence. The date of tumor recurrence was defined as the time at which the tumor recurrence was confirmed by radiological examination or AFP measurement. The recurrence-free survival (RFS) of patients was recorded.

### Statistical analysis

Data are summarized as the mean with standard deviation (SD) for continuous variables and percentages for discrete variables. Student’s *t* test and the Mann–Whitney *U* test were used for the comparison of continuous variables with a normal distribution and non-normal distribution, respectively. The chi-squared test was used for categorical variables. Survival analysis was performed using the Kaplan-Meier method and compared using the log-rank test. Receiver operating characteristic (ROC) analysis was used to determine the PLR cut-off value with the most significance in terms of predicting tumor recurrence after LT; the optimal PLR cut-off value was considered when the highest Youden index (sensitivity + specificity - 1) was presented. Cox proportional hazards regression was used to evaluate the risk factors of survival rates. Data were analyzed using SPSS 16.0 (SPSS Inc. Chicago, CA, USA). A *P* value <0.05 was considered statistically significant.

## Results

### Clinical characteristics of 343 patients received LT for HCC

**Fig. 1:**
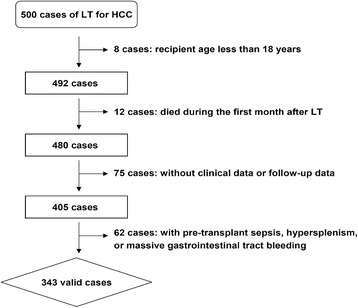
Schematic diagram of valid patients selection. A total of 500 cases of LT for HCC were performed, of these cases, 157 cases were excluded by exclusion criteria and 343 HCC patients were enrolled in this study

A total of 500 LT for HCC were performed, and 343 HCC patients were enrolled in this study after exclusion (Fig. 1). The mean age of recipients was 49.4 (19.0–71.0) years, and 308 (89.8%) cases were males and 35 (10.2%) were females. Of these patients, 320 (93.3%) were HBV infected, the pre-transplant MELD score was 13.0 ± 6.0, and LDLT was performed in 41 (12.0%) patients. Before LT, 52 (15.2%) patients received surgical tumor resections and 170 (49.6%) received interventional therapies. The mean follow-up period was 57.7 months, ranging from 33.5–156.0 months.

### Outcomes of patients divided according to the Milan and Hangzhou criteria

**Fig. 2:**
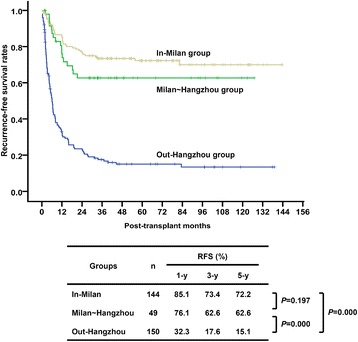
Outcomes of patients divided according to the Milan and Hangzhou criteria. The patients were divided into in-Milan group, Milan ~ Hangzhou group, and out-Hangzhou group. The 1-, 3-, and 5-year RFS of patients in the out-Hangzhou group were significantly worse than the other two groups. The RFS of the Milan ~ Hangzhou group were less than that of the in-Milan group, but no statistically significant difference was presented

Of these patients, 144 (42.0%) patients were within the Milan criteria (in-Milan group), 49 (14.3%) were beyond the Milan but within the Hangzhou criteria (Milan ~ Hangzhou group), and the remaining 150 patients (43.7%) were beyond the Hangzhou criteria (out-Hangzhou group). The 1-, 3-, and 5-year RFS of patients in the out-Hangzhou group were 32.3%, 17.6%, and 15.1%, respectively, significantly worse than the other two groups. The 1-, 3-, and 5-year RFS of the Milan ~ Hangzhou group were 76.1%, 62.6%, and 62.6%, respectively, less than the 85.1%, 73.4%, and 72.2% of the in-Milan group, respectively, but no statistically significant difference was presented (Fig. 2).

### Stratification of patients in the Milan ~ Hangzhou group by pre-transplant PLR values

The stratification analysis of patients in the Milan ~ Hangzhou group was performed using different PLR values. As described previously [12], we used the ROC curve to determine the PLR cut-off value with the most significance in predicting tumor recurrence after LT; the Youden index was highest when the PLR was 120 with a sensitivity and specificity of 61.6% and 62.7%, respectively. Therefore, we considered PLR = 120 as the optimal cut-off. After stratification, the 49 patients in the Milan ~ Hangzhou group were divided into the Milan ~ Hangzhou and PLR < 120 group (40 patients) and the Milan ~ Hangzhou and PLR ≥ 120 group (9 patients).

### Comparison of survival rates after stratification by PLR

**Fig. 3:**
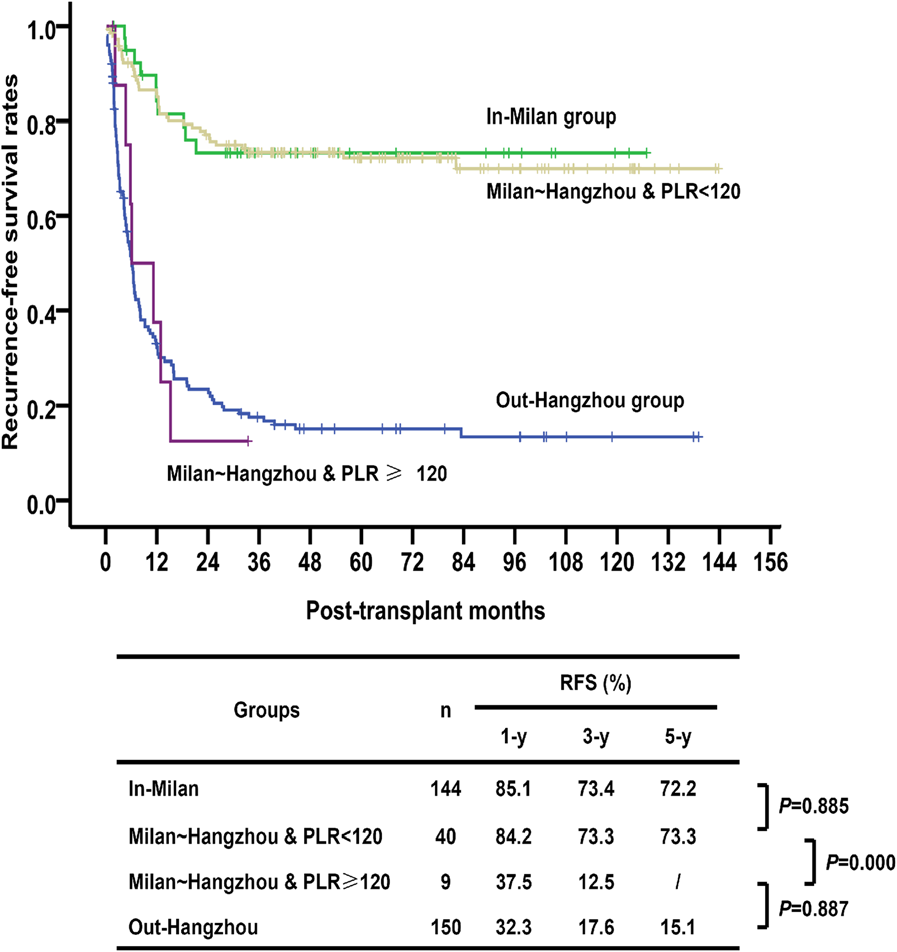
Comparison of survival rates after stratification by PLR. After stratification, the patients were divided into in-Milan group, Milan ~ Hangzhou & PLR < 120 group, Milan ~ Hangzhou & PLR ≥ 120 group, and out-Hangzhou group. The 1-, 3-, and 5-year RFS of the Milan ~ Hangzhou & PLR < 120 group were comparable with that of the in-Milan group. The Milan ~ Hangzhou & PLR ≥ 120 group showed poor outcomes which were similar to those of the out-Hangzhou group

As shown in Fig. 3, there were significant differences between the RFS of the two sub-groups divided according to PLR 120 (*P* = 0.000). The 1-, 3-, and 5-year RFS of the Milan ~ Hangzhou and PLR < 120 group were 84.2%, 73.3%, and 73.3%, respectively, comparable with 85.7%, 73.9%, and 72.8%, respectively, of the in-Milan group (*P* = 0.885). To our surprise, the Milan ~ Hangzhou and PLR ≥ 120 group showed very poor outcomes which were similar to those of the out-Hangzhou group, i.e. the 1-, 3-, and 5-year RFS were 37.5%, 12.5%, and 12.5% vs. 32.3%, 17.6%, and 15.1%, respectively (*P* = 0.887).

**Fig. 4:**
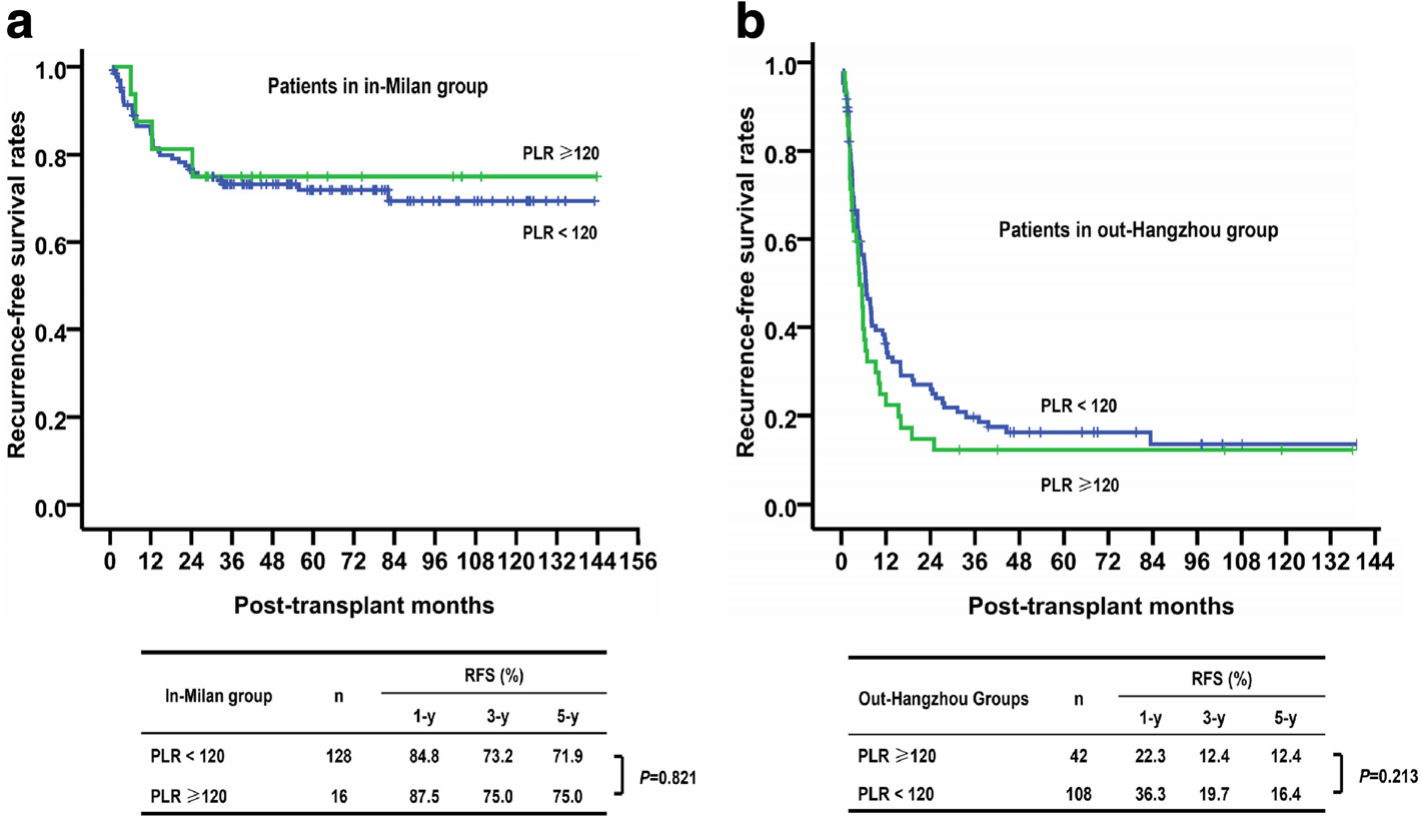
Differentiate value of PLR for patients of in-Milan or out-Hangzhou group. The patients of in-Milan or out-Hangzhou group were divided by PLR 120, there was no significant difference in RFS in either the in-Milan (Panel **a**) or out-Hangzhou group (Panel **b**)

We further stratified the patients fulfilling the Milan criteria or exceeding the Hangzhou criteria using PLR 120, but we could not find significant differences in RFS in either the in-Milan (Fig. 4a) or out-Hangzhou group (Fig. 4b). In addition, we also used different PLR cut-off values (90, 100, 110, 130, and 140) to do the survival analysis, but we failed to find any differentiate value of PLR in HCC patients fulfilling the Milan criteria (Additional file 1: Figure S1), or exceeding the Hangzhou criteria (Additional file 2: Figure S2).

### Establishment of the Hangzhou criteria & PLR method

Based on our present study, we developed the so-called “Hangzhou criteria & PLR method”: patients exceeding the Milan but fulfilling the Hangzhou criteria with PLR < 120, together with those fulfilling the Milan criteria, were regarded as fulfilling the Hangzhou criteria & PLR method, while patients exceeding the Milan but fulfilling the Hangzhou criteria with PLR **≥** 120 as well as patients exceeding the Hangzhou criteria should be regarded as exceeding the Hangzhou criteria & PLR method.

**Fig. 5:**
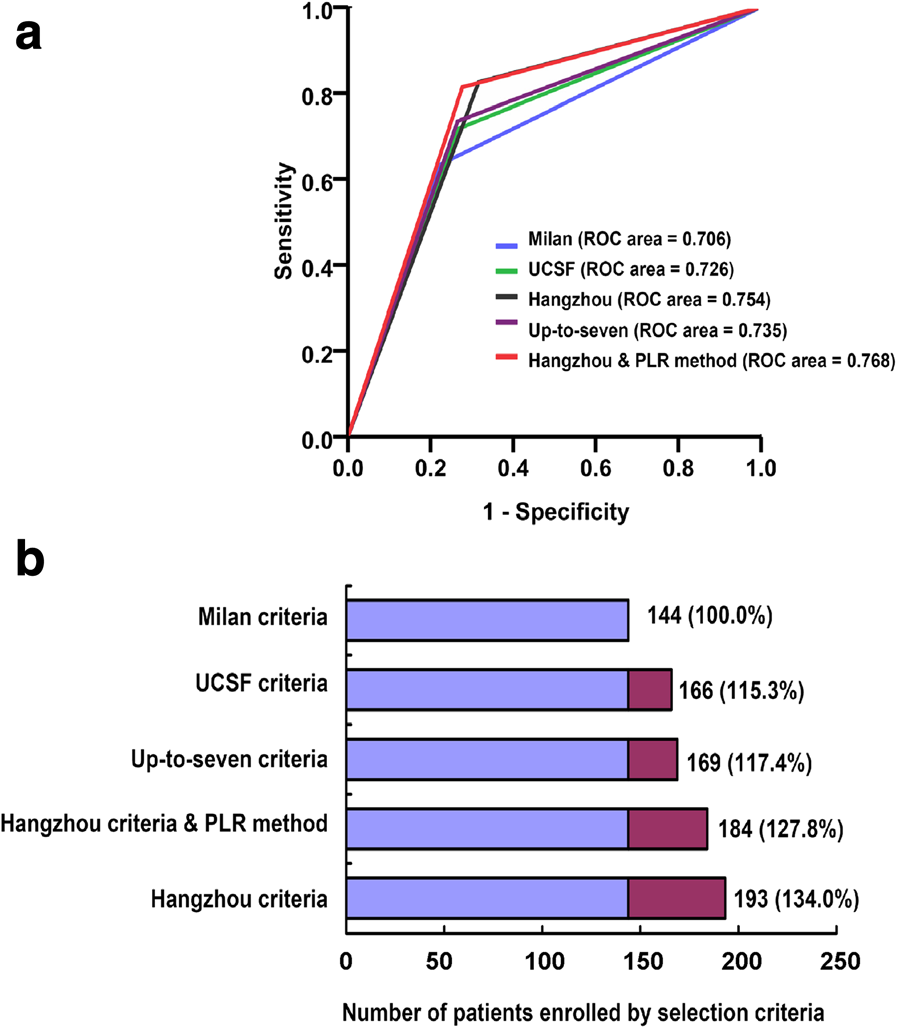
The comparison of different selection criteria. The ROC analysis showed that the ROC area of Hangzhou criteria & PLR method was higher than that of the current selection criteria including the Milan, UCSF, up-to-seven, and Hangzhou criteria (Panel **a**). The expansion of Hangzhou criteria & PLR method was lower than that of Hangzhou criteria, but higher than that of UCSF and up-to-seven criteria (Panel **b**)

Using the Hangzhou criteria & PLR method, the ROC analysis showed that the ROC area was 0.768 for RFS, higher than that of the current selection criteria including the Milan, UCSF, up-to-seven, and Hangzhou criteria (Fig. 5a).

In our cohort, compared to the Milan criteria, the UCSF, up-to-seven, Hangzhou criteria provided an expansion of 15.3% (*n* = 22), 17.4% (*n* = 25), and 34.0% (*n* = 49), respectively. The expansion of Hangzhou criteria & PLR method was 27.8% (*n* = 40), lower than that of Hangzhou criteria, but higher than that of UCSF and up-to-seven criteria (Fig. 5b).

### Tumor-related characteristics and clinical data in the Milan ~ Hangzhou and PLR < 120 and Milan ~ Hangzhou and PLR ≥ 120 groups

In order to identify an explanation for the poor outcomes in the Milan ~ Hangzhou and PLR ≥ 120 group, we evaluated the distribution of tumor-related characteristics and clinical data between the PLR ≥ 120 and < 120 groups of patients exceeding the Milan but fulfilling the Hangzhou criteria. As shown in Table 1, we found that a high proportion of patients with PLR ≥ 120 had a large tumor size of ≥ 7 cm, pre-transplant partial hepatectomy and interventional therapies. There was no difference in terms of recipient age, gender, MELD score, AFP level, HBV infection status, LDLT, microvascular invasion, tumor lesion number, or tumor differentiation. This result indicates that patients with PLR ≥ 120 tended to be associated with a large tumor size, pre-transplant partial hepatectomy and interventional therapies. These disparities may be an explanation for the poor prognosis of patients with PLR ≥ 120.

**Table 1:** Comparison of tumor-related characteristics and clinical data between the Milan ~ Hangzhou and PLR < 120 and Milan ~ Hangzhou and PLR ≥ 120 groups

Variables	Milan ~ Hangzhou group		*P* value
	PLR < 120 (*n* = 40)	PLR ≥ 120 (*n* = 9)	
Age (years)	50.5 ± 8.4	51.3 ± 11.2	0.802
Gender (Male)	36 (90.0%)	7 (77.8%)	0.302
MELD score	12.6 ± 4.6	10.4 ± 2.5	0.062
AFP (ng/ml)	675.1 ± 1282.9	1248.0 ± 2808.1	0.565
HBV infection	37 (92.5%)	7 (77.8%)	0.224
Blood cell count (*10^9^/L)			
Neutrophil	2.1 ± 1.4	2.4 ± 2.3	0.740
Lymphocyte	1.1 ± 0.7	0.7 ± 0.4	0.089
Platelet	84.0 ± 64.4	153.2 ± 108.5	0.098
Pre-LT treatment			
Surgical resection	5 (12.5%)	4 (44.4%)	0.046
Interventional therapy	18 (45.0%)	8 (88.9%)	0.026
Types of LT			1.000
LDLT	3 (7.5%)	0 (0.0%)	
DDLT	37 (92.5%)	9 (100.0%)	
Solitary tumor	19 (47.5%)	6 (66.7%)	0.463
Maximal tumor **≥** 7 cm	7 (17.5%)	5 (55.6%)	0.029
Well-moderate differentiation	28 (70.0%)	8 (88.9%)	0.412
Microvascular invasion	8 (20.0%)	1 (11.1%)	1.000

### Univariate and multivariate analysis of risk factors for RFS of patients in Milan ~ Hangzhou group

The univariate Cox regression analysis showed that pre-transplant PLR ≥ 120, AFP ≥ 200 ng/ml and pre-LT surgical resection were risk factors of RFS of patients in Milan ~ Hangzhou group. Based on multivariate Cox regression analysis, pre-transplant PLR ≥ 120 (hazard ratio [HR] = 5.194, *P* = 0.020) and AFP ≥ 200 ng/ml (HR = 4.313, *P* = 0.004) were confirmed as independent risk factors of RFS of HCC patients in Milan ~ Hangzhou group (Table 2).

**Table 2:** Univariate and multivariate analysis of risk factors for RFS of patients in Milan ~ Hangzhou group

Characteristics	Univariate	Multivariate	
	*P*	*P*	HR (95% CI)
Recipient			
Age ≥ 60 years	0.093		
Gender (Male)	0.067		
MELD score ≥ 20	0.517		
HBV infection	0.852		
Types of LT: LDLT	0.219		
PLR ≥ 120	0.000	0.020	5.194 (1.293 ~ 20.865)
Tumor-related			
Pre-LT treatment			
Surgical resection	0.023	0.919	1.082 (0.236 ~ 4.966)
Interventional therapy	0.539		
AFP ≥ 200 ng/ml	0.000	0.004	4.313 (1.591 ~ 11.695)
Solitary tumor	0.118		
Maximal tumor ≥ 7 cm	0.743		
Well-moderate differentiation	0.374		
Microvascular invasion	0.208		

## Discussion

Since the induction of the Milan criteria by Mazzaferro et al. in 1996, excellent clinical outcomes after LT have been achieved for HCC patients fulfilling the Milan criteria [5, 13]. In the following decades, the selection criteria were expanded effectively and safely [6, 7]. The current selection criteria are based solely or mainly on a pre-transplant radiological examination, which places no consideration on tumor biological behavior and is limited in detecting small tumor lesions. Radiological imaging has been reported to be insufficiently accurate and always underestimates the real tumor status [14]. To avoid the bias caused by radiological imaging, many studies have been performed to identify biological markers which can be used in combination with the current selection criteria, including AFP [15], matrix metalloproteinase-9 [16], osteopontin [17], and albumin mRNA [18]. For example, Sieghart et al. indicated that serum osteopontin is an independent predictor of RFS in patients beyond the Milan criteria, and the authors suggested that osteopontin staining in liver biopsies should be performed at the time of LT, especially for the candidates exceeding the Milan criteria [17]. Recently, Xu et al. studied a large cohort of Chinese HCC patients and stratified patients fulfilling the Hangzhou criteria into type A (tumor burden ≤8 cm or tumor burden >8 cm, but with AFP ≤100 ng/mL and well to moderate differentiation) and type B (tumor burden >8 cm, but AFP between 100 and 400 ng/mL and well to moderate differentiation). These authors found that Hangzhou type B was associated with a relatively poor prognosis, and suggested that Hangzhou type B should be regarded as a relative contraindication for LT considering the shortage of organs [9].

In our study, the pre-transplant PLR was employed in combination with Hangzhou criteria to select HCC candidates for LT. In recent years, studies have confirmed that elevated systemic inflammation predicts poor prognosis in various kinds of cancers, including HCC [19]. The tumor can stimulate the inflammatory process, and inflammatory cells can promote angiogenesis, tumor proliferation, and metastasis by complicated molecular mechanisms [20, 21]. Various indices have been used to evaluate systemic inflammation, including the PLR, neutrophil-to-lymphocyte ratio [22], and modified Glasgow prognostic score [23]. All these indices can be easily calculated based on routinely performed blood tests in the clinical work-up. For PLR, it can be used to predict the prognosis of HCC patients receiving transarterial chemoembolization [24], thermal ablation [25], or partial hepatectomy [26]. Our previous work also confirmed that pre-transplant elevated PLR was associated with a high proportion of multiple tumors, large tumor size, micro- and macrovascular invasion, and predicted post-transplant tumor recurrence [12].

Our present study identified that HCC patients exceeding the Milan but fulfilling the Hangzhou criteria could be stratified effectively by the pre-transplant PLR value. After stratification, the patients exceeding the Milan but fulfilling the Hangzhou criteria with PLR ≥ 120 showed poor outcomes, which were very similar to those of patients exceeding the Hangzhou criteria, indicating that PLR ≥ 120 effectively excludes high-risk tumor recurrence patients from candidates exceeding the Milan but fulfilling the Hangzhou criteria. The prognosis of patients exceeding the Milan but fulfilling the Hangzhou criteria with PLR < 120 was almost the same as patients fulfilling the Milan criteria. Furthermore, the multivariate analysis confirmed that PLR ≥ 120 was an independent risk factor of RFS of HCC patients exceeding the Milan but fulfilling the Hangzhou criteria. Consistent with previous studies [27], PLR failed to predict post-transplant tumor recurrence for patients fulfilling the Milan criteria. Our results show that patients fulfilling the Milan criteria can obtain satisfactory outcomes whether elevated pre-transplant PLR is present or not. In line with our estimation, patients exceeding the Hangzhou criteria were associated with poor prognosis regardless of their PLR status.

According to this study, we propose the Hangzhou criteria & PLR method. In this method, patients exceeding the Milan but fulfilling the Hangzhou criteria with PLR < 120 should be regarded homogenously as those fulfilling the Milan criteria; similarly, patients exceeding the Hangzhou criteria, and patients who exceed the Milan but fulfill the Hangzhou criteria with PLR ≥ 120 should be regarded as contraindicated for LT. The ROC analysis confirmed the feasibility of this new method and demonstrated its superiority to other selection criteria. Compared to the Milan criteria, 40 (27.8%) more patients were provided the option of LT by the Hangzhou criteria & PLR method without any sacrifice in prognosis.

One limitation of our study was the small number of enrolled patients, and all these recipients were Chinese citizens and majority of them were hepatitis B virus infected. Further studies based on a larger, multi-center cohort need to be carried out to verify our conclusions. The advantage of our study was that the PLR value we used can be obtained easily and conveniently from the routinely performed complete blood count, in contrast to other molecular or genetic markers or complicated mathematical predictive models.

## Conclusions

In order to maximize recipient benefit and make full use of scarce liver grafts, we suggest that pre-transplant PLR can be combined with the Hangzhou criteria to select LT candidates more carefully. The Hangzhou criteria combined with the pre-transplant PLR can accurately exclude high-risk tumor recurrence recipients; this approach expands the Milan criteria effectively without any sacrifice.

## Additional files

Additional file 1: Figure S1. RFS of patients fulfilling the Milan criteria stratified by different pre-transplant PLR values. For patients who fulfilled the Milan criteria, the RFS was comparable after stratification by different PLR cut-off values (90, 100, 110, 130, and 140). (TIF 738 kb)
Additional file 2: Figure S2. Differentiate value of PLR for patients exceeding the Hangzhou criteria. There was no significant difference in RFS for patients exceeding the Hangzhou criteria after stratification by different PLR cut-off values (90, 100, 110, 130, and 140). (TIF 52 kb)

## Abbreviations

AFP: α-fetoprotein
DDLT: deceased donor liver transplantation
HBV: hepatitis B virus
HCC: hepatocellular carcinoma
LDLT: living donor liver transplantation
LT: liver transplantation
MELD: model of end-stage liver disease
PLR: platelet to lymphocyte ratio
RFS: recurrence-free survival
ROC: receiver operating characteristic
SD: standard deviation
UCSF: University of California San Francisco
