# Supplementary figures and images for: Expansion of the Milan criteria without any sacrifice: combination of the Hangzhou criteria with the pre-transplant platelet-to-lymphocyte ratio
Source: BMC Cancer. 2017 Jan 5;17:14. doi: 10.1186/s12885-016-3028-0 (PMC5216555; doi:10.1186/s12885-016-3028-0)

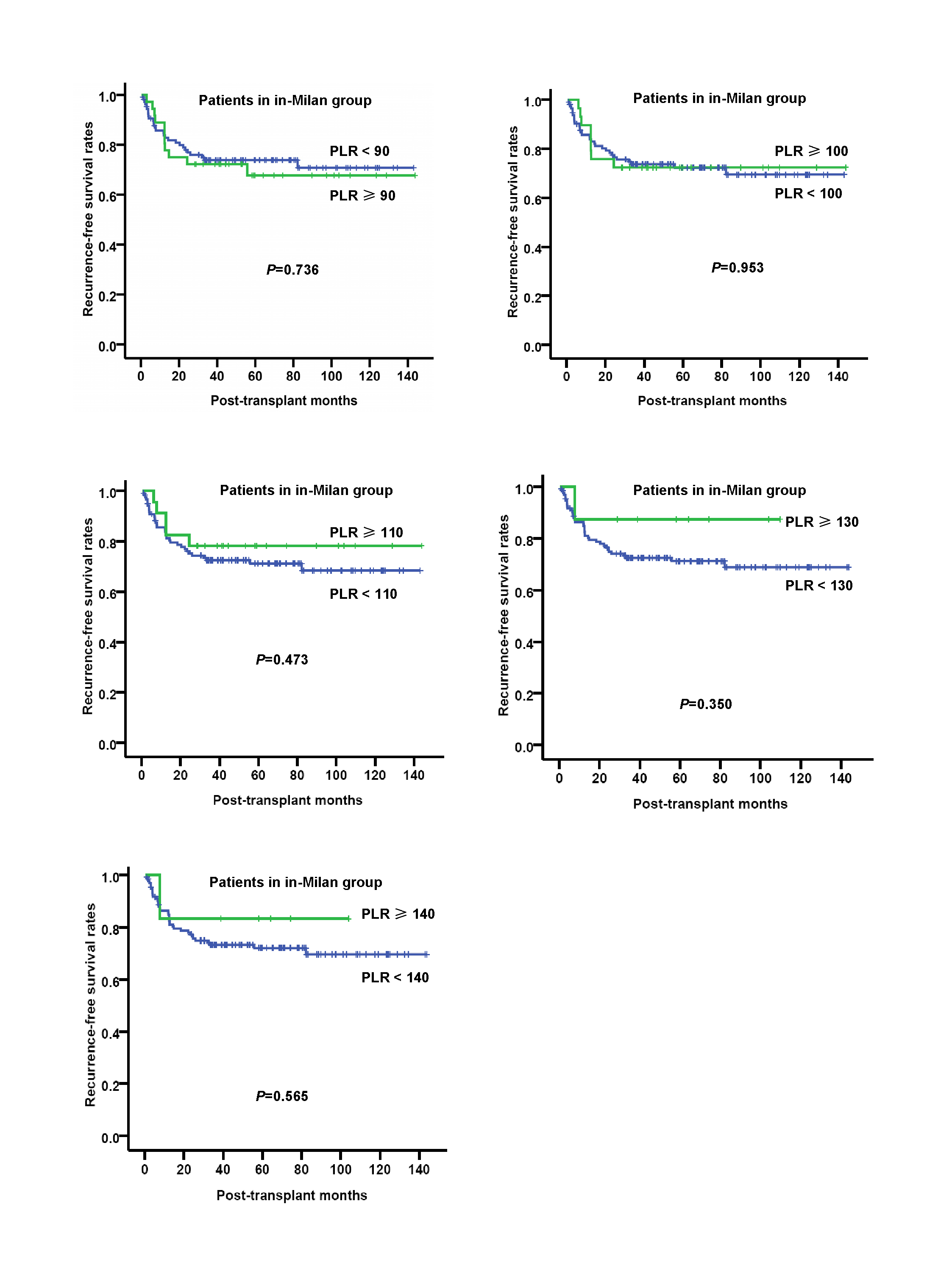

Supplement: Additional file 1: Figure S1. — RFS of patients fulfilling the Milan criteria stratified by different pre-transplant PLR values. For patients who fulfilled the Milan criteria, the RFS was comparable after stratification by different PLR cut-off values (90, 100, 110, 130, and 140). (TIF 738 kb) [file 12885_2016_3028_MOESM1_ESM.tif]
